# Development of a New Reverse Genetics System for Ebola Virus

**DOI:** 10.1128/mSphere.00235-21

**Published:** 2021-05-05

**Authors:** Tianyu Gan, Dihan Zhou, Yi Huang, Shuqi Xiao, Ziyue Ma, Xiaoyou Hu, Yimin Tong, Huimin Yan, Jin Zhong

**Affiliations:** a Unit of Viral Hepatitis, CAS Key Laboratory of Molecular Virology and Immunology, Institut Pasteur of Shanghai, Center for Biosafety Mega-Science, Chinese Academy of Sciences, Shanghai, China; b The Joint Laboratory for Translational Precision Medicine, Guangzhou Women and Children’s Medical Center, Guangzhou, China, and Wuhan Institute of Virology, Chinese Academy of Sciences, Wuhan, China; c Wuhan National Biosafety Laboratory, Center for Biosafety Mega-Science, Chinese Academy of Sciences, Wuhan, China; d Mucosal Immunity Research Group, State Key Laboratory of Virology, Wuhan Institute of Virology, Chinese Academy of Sciences, Wuhan, China; e University of Chinese Academy of Sciences, Beijing, China; f ShanghaiTech University, Shanghai, China; University of Michigan-Ann Arbor

**Keywords:** Ebola virus, filovirus, negative-stranded RNA virus, reverse genetics

## Abstract

Ebola virus (EBOV) is a highly pathogenic negative-stranded RNA virus that has caused several deadly endemics in the past decades. EBOV reverse genetics systems are available for studying live viruses under biosafety level 4 (BSL-4) or subviral particles under BSL-2 conditions. However, these systems all require cotransfection of multiple plasmids expressing viral genome and viral proteins essential for EBOV replication, which is technically challenging and unable to naturally mimic virus propagation using the subviral particle. Here, we established a new EBOV reverse genetics system only requiring transfection of a single viral RNA genome into an engineered cell line that stably expresses viral nucleoprotein (NP), viral protein 35 (VP35), VP30, and large (L) proteins and has been fine-tuned for its superior permissiveness for EBOV replication. Using this system, subviral particles expressing viral VP40, glycoprotein (GP), and VP24 could be produced and continuously propagated and eventually infect the entire cell population. We demonstrated the authentic response of the subviral system to antivirals and uncovered that the VP35 amount is critical for optimal virus replication. Furthermore, we showed that fully infectious virions can be efficiently rescued by delivering the full-length EBOV genome into the same supporting cell, and the efficiency is not affected by genome polarity or virus variant specificity. In summary, our work provides a new tool for studying EBOV under different biosafety levels.

**IMPORTANCE** Ebola virus is among the most dangerous viral pathogens, with a case fatality rate of up to 90%. Since 2013, the two largest and most complex Ebola outbreaks in Africa have revealed the lack of investigation on this notorious virus. A reverse genetics system is an important tool for studying viruses by producing mutant viruses or generating safer and convenient model systems. Here, we developed an EBOV life cycle modeling system in which subviral particles can spontaneously propagate in cell culture. In addition, this system can be employed to rescue infectious virions of homologous or heterologous EBOV isolates using either sense or antisense viral RNA genomes. In summary, we developed a new tool for EBOV research.

## Abstract (precis)

Ebola virus is among the most dangerous viral pathogens, with a case fatality rate of up to 90%. Since 2013, the two largest and most complex Ebola outbreaks in West Africa have revealed the lack of investigation on this notorious virus.

## INTRODUCTION

Ebola virus (EBOV), a highly pathogenic agent, has caused over 11,000 deaths in the 2013 to 2016 outbreak in West Africa (http://apps.who.int/gho/data/view.ebola-sitrep.ebola-summary-latest?lang=en) and over 2,200 deaths in the 2018 to 2020 outbreak in the Democratic Republic of the Congo (DRC) (https://www.who.int/emergencies/situations/Ebola-2019-drc-), even though more than 300,000 people have been vaccinated with the recently approved vaccine (https://www.fda.gov/news-events/press-announcements/first-fda-approved-vaccine-prevention-ebola-virus-disease-marking-critical-milestone-public-health). Belonging to the family of *Filoviridae*, EBOV has a nonsegmented negative-sense RNA genome of approximately 19 kb, which encodes 7 virion-associated structural proteins, including nucleoprotein (NP), viral protein 35 (VP35), VP40, glycoprotein (GP), VP30, VP24, and large protein (L) that is the RNA-dependent RNA polymerase (RdRp) (1, 2). Of these 7 viral proteins, NP, VP35, VP30, and L assemble into a ribonucleoprotein (RNP) complex on viral RNA and are sufficient for catalyzing EBOV genome replication and transcription (3). While they may impact virus replication (4, 5), viral matrix proteins VP40 and VP24 are dispensable for viral replication and transcription but rather essential for virion assembly, along with GP (6, 7).

While promising progress has been achieved in clinical trials of antibody- or small molecule-based antiviral therapies (8, 9), their real-world applications remain challenging. Also, novel EBOV variants and other related highly pathogenic filoviruses continue to emerge and impose potential threats to public health worldwide, necessitating more and deeper dissection of these viruses (10). The requirement of a biosafety level 4 (BSL-4) laboratory to handle live EBOV, however, has limited the research force on this notorious virus. To circumvent this hurdle, life cycle modeling systems to study virological features and screen antivirals outside BSL-4 have been developed (11), such as the tetracistronic transcription- and replication-competent virus-like particle (trVLP) system (12) and EbolaΔVP30 virus (13), which can be used to model almost all aspects of the virus life cycle under BSL-2/3 conditions. Tetracistronic trVLP is an advanced version of previous minigenomic trVLP (14, 15) to increase specificity and productivity and is produced with tetracistronic subgenome (SG) expressing viral gene VP40, GP, and VP24, which are required for virion assembly as well as a reporter. However, current EBOV reverse genetics systems depend on cotransfection of multiple plasmids, including 4 plasmids expressing NP, VP35, VP30, and L required for initial transcription and replication of the negative-sense viral genome, a plasmid encoding the viral genome under the T7 promoter, and a plasmid expressing the T7 RNA polymerase. Furthermore, the rescued trVLP can only produce transient and incomplete infection in wild-type cells and cannot be propagated unless viral NP, VP35, VP30, and L are continuously provided. In addition, HEK293T cells, which are often used in the cotransfection experiment for their efficient transfectability, are found suboptimal for trVLP infection and may require the expression of additional critical host factors (12).

In this study, we established a novel EBOV reverse genetics system which only requires transfection of a single element (viral RNA genome) into a helper cell line that had been engineered and optimized for its superior ability to support EBOV replication and propagation. Subgenomic trVLP can be readily produced, propagated to the entire cell population, and reach high titers after delivery of the EBOV subgenome expressing viral proteins required for virus assembly (VP40, GP, and VP24) into this helper cell line. Furthermore, fully infectious virions of homologous and heterologous EBOV isolates can also be efficiently rescued by delivering the full-length genome into the helper cells. Our work not only provides a new tool for studying EBOV under different biosafety level conditions but also may render a new strategy to develop reverse genetics systems for other negative-stranded RNA viruses.

## RESULTS

### Construction of a helper cell line that efficiently supports EBOV-trVLP propagation.

**FIG 1:**
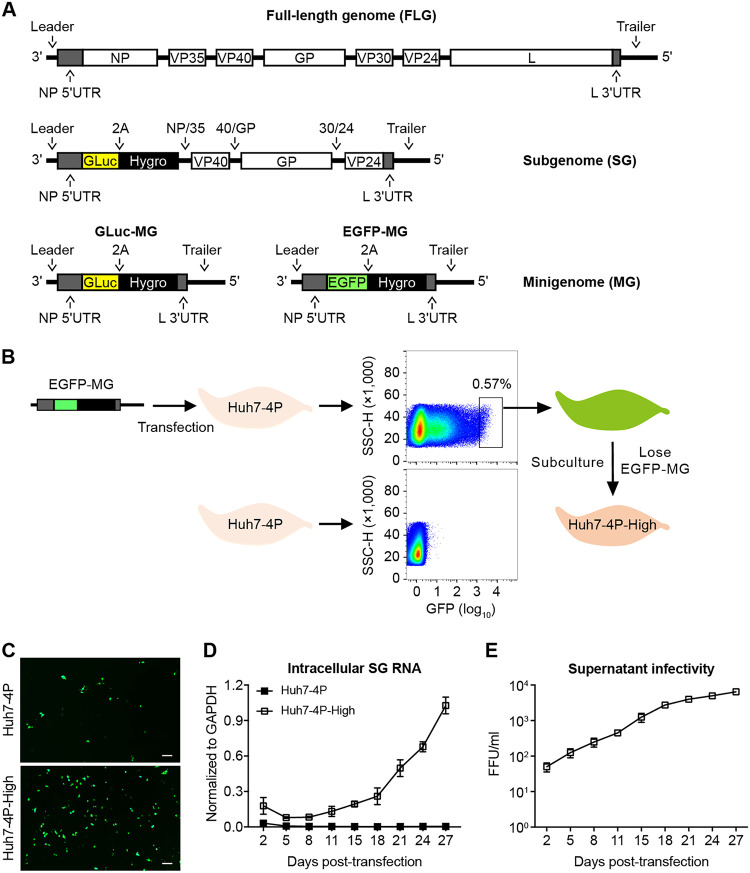
Generation and propagation of EBOV-trVLP on permissive cells. (A) Schematics of EBOV constructs used in this study, including full-length genome (FLG), subgenome (SG) containing VP40, GP, and VP24, as well as two versions of minigenome (MG) containing GLuc-hygromycin (GLuc-MG) or EGFP-hygromycin (EGFP-MG). (B) Protocol to establish Huh7-4P-High cells that are more permissive for SG replication and trVLP propagation. (C) Huh7-4P and Huh7-4P-High cells that had been transfected with EBOV EGFP-MG RNA for 48 h were analyzed for the GFP expression by fluorescence microscopy. Scale bar, 100 μm. (D and E) Huh7-4P and Huh7-4P-High cells were transfected with Mayinga-SG RNA and cultured for 27 days. Intracellular SG RNA levels (D) and extracellular trVLP titers (E) were measured at the indicated time points by reverse transcriptase quantitative PCR (RT-qPCR) and titration assay, respectively. The data shown in panels D and E are results from 1 of 2 independent experiments and are presented as the mean ± SD of *n* = 2 technical replicates. The data of another experiment are shown in Fig. S2A and B.

We previously established a Huh7-based cell line (Huh7-4P) stably expressing EBOV NP, VP35, VP30, and L proteins that are essential for viral transcription and replication (16). By using this cell line, we successfully established a stably replicating EBOV minigenome (MG) replicon that contains the viral *cis* elements (leader, trailer, and untranslated regions [UTRs] of NP and L) as well as a self-cleaving fusion protein of *Gaussia* luciferase reporter and a hygromycin-resistant selectable marker (GLuc-Hygro) (16) (Fig. 1A). Here, we first sought to determine whether Huh7-4P cells support the replication of a Yambuku-Mayinga isolate-based tetracistronic subgenome (SG) consisting of viral gene VP40, VP24, unedited GP, and GLuc-Hygro (Fig. 1A). However, the percentage of VP40-positive cells and EBOV-SG RNA levels (intracellular and extracellular) decreased continuously after transfection of *in vitro*-transcribed EBOV-SG RNA into the Huh7-4P cells (Fig. S1 in the supplemental material), suggesting that the Huh7-4P cell does not efficiently support the SG replication.

10.1128/mSphere.00235-21.1 FIG S1 Huh7-4P cells do not efficiently support replication of EBOV-SG. Huh7 and Huh7-4P cells were transfected with Mayinga-SG RNA. VP40 immunofluorescence (A), intracellular SG RNA levels (B), and extracellular SG RNA levels (C) were measured at the indicated time points. Scale bars in panel A, 100 μm. Data shown in panels B and C are results from 2 independent experiments and are presented as the mean ± SD of *n* = 2 biological replicates. Download FIG S1, PDF file, 0.8 MB. Copyright © 2021 Gan et al. 2021 Gan et al. https://creativecommons.org/licenses/by/4.0/ This content is distributed under the terms of the Creative Commons Attribution 4.0 International license.

10.1128/mSphere.00235-21.2 FIG S2 Huh7-4P-High cells confer a better ability than Huh7-4P cells to support replication of EBOV-SG and propagation of trVLP. (A and B) Huh7-4P and Huh7-4P-High cells were transfected with Mayinga-SG RNA and cultured for 24 days. Intracellular SG RNA levels (A) and extracellular trVLP titers (B) were measured at the indicated time points by RT-qPCR and titration assay, respectively. This experiment is a biological replicate of another experiment described in Fig. 1D and E, and the data are presented as the mean ± SD of *n* = 2 technical replicates. (C) Huh7-4P and Huh7-4P-High cells were transfected with Mayinga-SG RNA and cultured for 27 days. VP40 immunofluorescence was measured by fluorescence microscopy at the indicated time points. (D) Huh7, Huh7-4P, and Huh7-4P-High cells that had been infected with trVLP at an MOI of 0.1 for 3 days were analyzed by VP40 immunofluorescence. Scale bars, 100 μm. Download FIG S2, PDF file, 1.5 MB. Copyright © 2021 Gan et al. 2021 Gan et al. https://creativecommons.org/licenses/by/4.0/ This content is distributed under the terms of the Creative Commons Attribution 4.0 International license.

Given that the Huh7-4P cell line is a mixed cell population possessing different stoichiometry of the 4 viral proteins in each cell, which has been shown to be critical for EBOV replication (14, 17, 18), we designed an approach to select a cell from the Huh7-4P pool that efficiently supports EBOV-SG replication. Huh7-4P cells were transfected with an EBOV-MG consisting of an enhanced green fluorescent protein (EGFP-MG) (Fig. 1A). The cells with strong GFP signals were sorted out by flow cytometry (Fig. 1B) and then cultured continuously for 1 month, leading to complete loss of the GFP signals and EBOV-MG RNA in the cells (denoted Huh7-4P-High) (data not shown).

To assess the ability of Huh7-4P-High cells to support EBOV replication, EGFP-MG RNA was transfected into Huh7-4P-High or Huh7-4P cells. As shown in Fig. 1C, Huh7-4P-High cells indeed displayed an improved ability to support the EGFP-MG replication. Next, we assessed the ability of Huh7-4P-High cells to support EBOV-SG replication. Transfection of EBOV-SG RNA into Huh7-4P-High cells led to a gradual increase of intracellular SG RNA level (Fig. 1D and Fig. S2A), extracellular trVLP titers (Fig. 1E and Fig. S2B), and percentage of VP40-positive cells (Fig. S2C), indicating efficient EBOV-SG replication as well as trVLP spread. Consistently, trVLP infection in Huh7-4P-High cells was more efficient than that in Huh7-4P cells (Fig. S2D).

**FIG 2:**
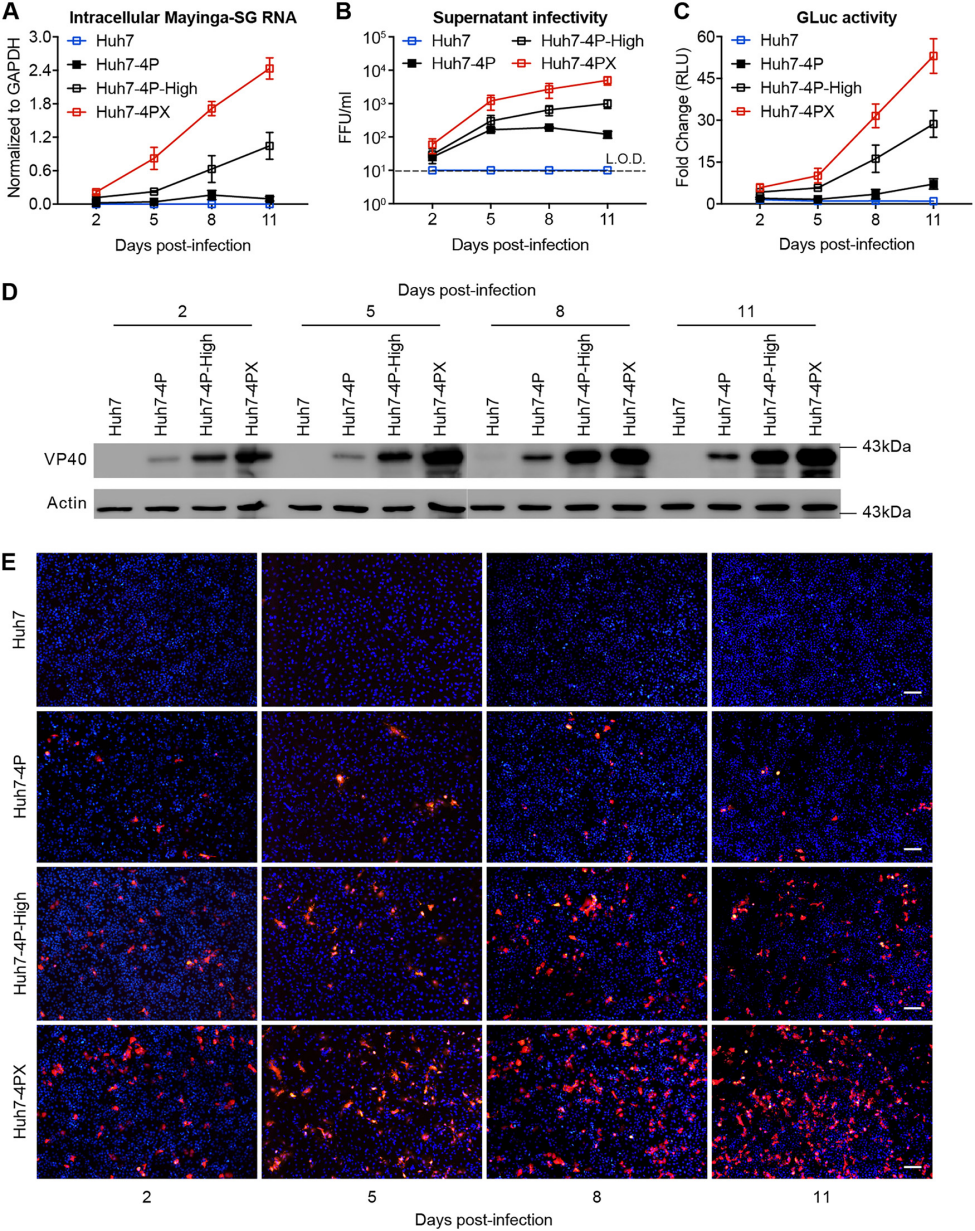
Huh7-4PX cells are highly efficient in supporting EBOV-SG replication and trVLP propagation. Huh7, Huh7-4P, Huh7-4P-High, and Huh7-4PX cells were infected with trVLP at an MOI of 0.1 and cultured for 11 days. Intracellular SG RNA level (A), extracellular trVLP titers (B), luciferase activities (C), VP40 Western blotting (D), and VP40 immunofluorescence (E) were measured at the indicated time points. The limit of detection (L.O.D.) is indicated by a dashed line in panel B. Scale bars in panel E, 100 μm. Data shown in panels A to C are the results from 2 independent experiments and are presented as the mean ± SD of *n* = 2 biological replicates.

To further improve the permissiveness for EBOV-trVLP propagation, we subcloned Huh-4P-High cells (Fig. S3A). Seven cell clones were generated, among which clone 1 (denoted Huh7-4PX) showed a strong permissiveness for EBOV-trVLP propagation and EBOV-SG replication (Fig. S3A and S3B) and thus was selected for the following studies. Of note, we confirmed that no residual EGFP-MG RNA previously introduced to enrich permissive cells was detected in Huh7-4P-high cells and in each of these subclones (Fig. S3C). Next, we assessed the ability of Huh7-4PX cells to support trVLP propagation. Naive Huh7, Huh7-4P, Huh7-4P-High, and Huh7-4PX cells were inoculated with trVLP. As shown in Fig. 2, intracellular SG RNA level (Fig. 2A), extracellular trVLP titers (Fig. 2B), GLuc activity (Fig. 2C), and VP40 protein expression level (Fig. 2D and E) increased most rapidly and reached the highest level on day 11 postinfection in Huh7-4PX cells, demonstrating that Huh7-4PX cells indeed possess a superior ability to support EBOV replication and propagation.

10.1128/mSphere.00235-21.3 FIG S3 Subcloning of Huh7-4P-High cells to obtain a more permissive cell clone Huh7-4PX. Huh7-4P-High and its derivative monoclonal cells were infected with trVLP at an MOI of 0.1. VP40 immunofluorescence (A), intracellular SG RNA levels (B), and intracellular MG RNA levels (C) were measured on day 3 postinfection. The limit of detection (L.O.D.) was indicated by a dashed line in panel C. Scale bars in panel A, 100 μm. Data in panels B and C are presented as the mean ± SD of *n* = 2 biological replicates. Download FIG S3, PDF file, 1.0 MB. Copyright © 2021 Gan et al. 2021 Gan et al. https://creativecommons.org/licenses/by/4.0/ This content is distributed under the terms of the Creative Commons Attribution 4.0 International license.

### Characterization of Huh7-4PX cell-derived trVLP.

**FIG 3:**
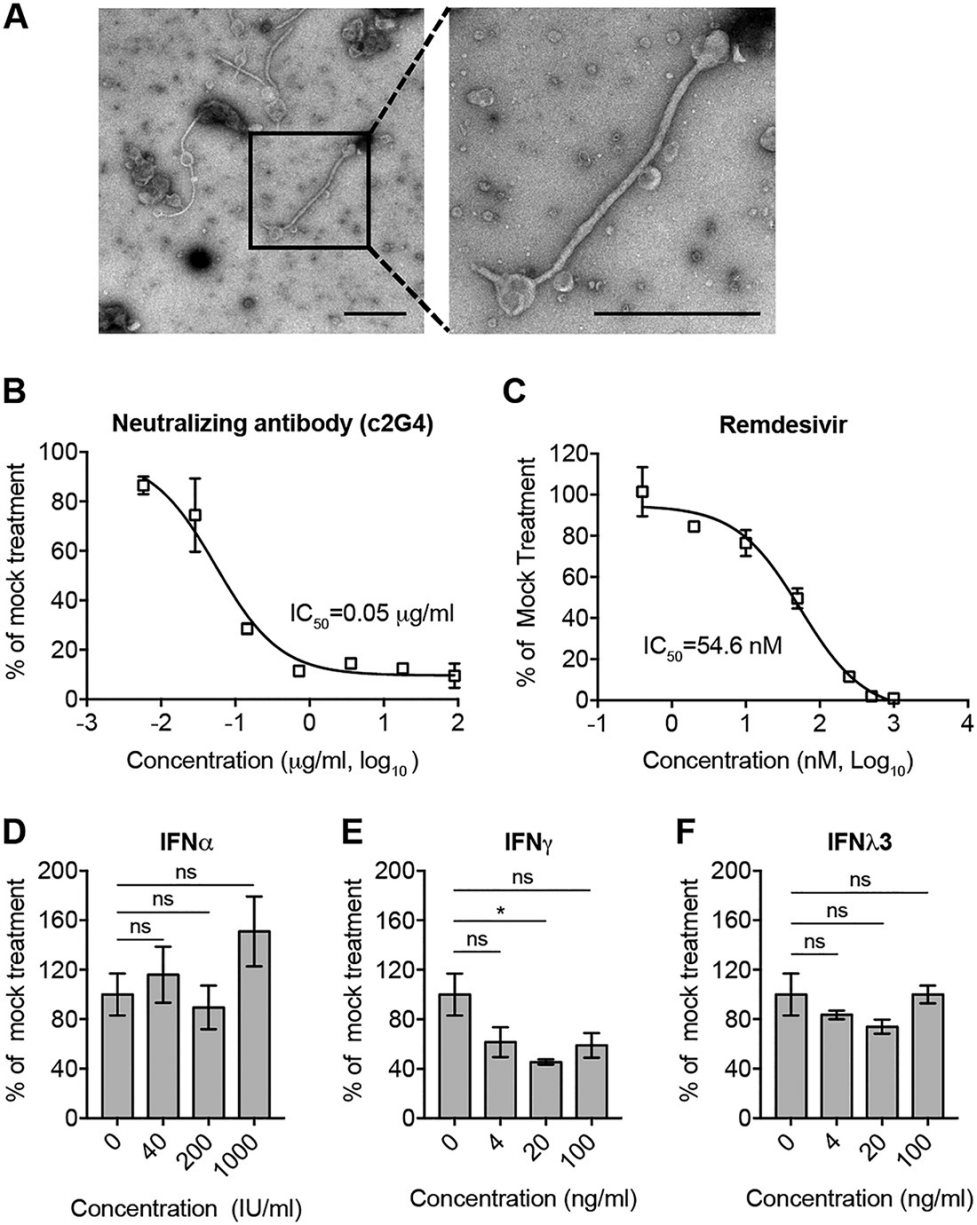
Characterization of Huh7-4PX cell-derived EBOV-trVLP. (A) trVLP from culture supernatant was enriched and purified through ultracentrifugation and analyzed by TEM. Scale bar, 1 μm. (B) trVLP was incubated with neutralizing antibody c2G4 at the indicated concentration for 1 h at 37°C and used to infect Huh7-4PX cells for 2 days. VP40 immunofluorescence foci number of each group was used to calculate the IC_50_ with GraphPad software. (C) trVLP-infected Huh7-4PX cells were treated with remdesivir at the indicated concentration for 3 days. Intracellular SG RNA level of each group was used to calculate the IC_50_ with GraphPad software. (D to F) Huh7-4PX cells were pretreated with the indicated doses of IFN-α (D), IFN-γ (E), or IFN-λ3 (F) for 12 h and then infected with trVLP for 60 h. Intracellular SG RNA level was measured by RT-qPCR. Data in panels B to F are presented as the mean ± SD of *n* = 2 biological replicates. *, *P* < 0.05; ns, not significant.

To characterize the features of EBOV-trVLP propagated in Huh7-4PX cells, we first analyzed trVLP morphologically. Huh7-4PX cells were infected with EBOV-trVLP at a multiplicity of infection (MOI) of 0.1 for 12 days. trVLP from 120 ml of culture supernatants was recovered by ultracentrifugation and then analyzed by transmission electron microscopy (TEM). As shown in Fig. 3A, trVLP displayed filamentous morphology as previously reported for infectious EBOV (19).

Next, we determined whether the produced EBOV-trVLP in Huh7-4PX cells can be inhibited by two previously reported countermeasures. Neutralizing antibody c2G4, a component of ZMapp, efficiently inhibited trVLP infection at 50% inhibitory concentration (IC_50_) of 0.05 μg/ml (Fig. 3B), which was comparable to its inhibition on infectious virus (20). Remdesivir, a nucleotide analog, also displayed a similar inhibitory effect on trVLP compared to that on EBOV (21) (Fig. 3C). We then evaluated the anti-EBOV effect of type I, II, and III interferons (IFNs), whose effect against EBOV remain controversial from previous studies (22–25). As shown in Fig. 3D to F, only IFN-γ slightly inhibited trVLP infection, whereas neither IFN-α nor IFN-λ3 had any effect.

**FIG 4:**
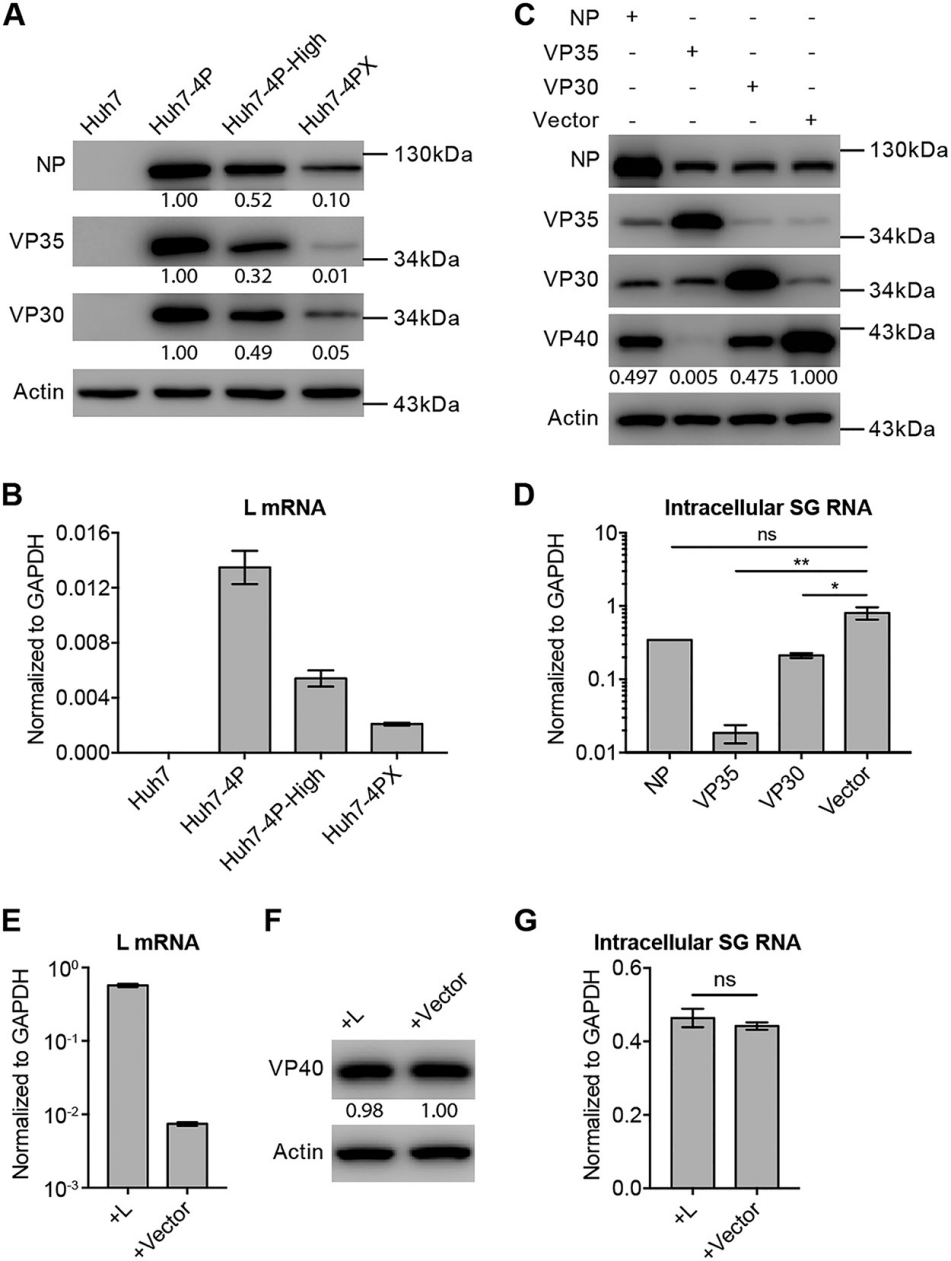
VP35 expression level is critical for Huh7-4PX to support EBOV-SG replication. (A) NP, VP35, and VP30 protein levels in Huh7, Huh7-4P, Huh7-4P-High, and Huh7-4PX cells were analyzed by Western blotting. (B) L mRNA levels in Huh7, Huh7-4P, Huh7-4P-High, and Huh7-4PX cells were determined by RT-qPCR. (C to D) Huh7-4PX cells that had been transduced with NP-, VP35-, or VP30-encoding lentiviruses were infected with trVLP at an MOI of 0.1 for 72 h. NP, VP35, VP30, and VP40 protein levels (C) and intracellular SG RNA levels (D) were analyzed by Western blotting and RT-qPCR, respectively. (E to G) Huh7-4PX cells that had been transfected with L-encoding pcDNA3.1 plasmid were infected with trVLP at an MOI of 0.1 for 72 h. L mRNA (E), VP40 protein levels (F), and intracellular SG RNA levels (G) were analyzed by Western blotting and RT-qPCR, respectively. Data in panels B, D, E, and G are presented as the mean ± SD of *n* = 2 biological replicates. **, *P* < 0.01; *, *P* < 0.05; ns, not significant.

To probe into why the Huh7-4PX cell can more efficiently support EBOV-SG replication than its parental cells, we measured the level of NP, VP35, and VP30 proteins and L mRNA (due to lack of anti-L antibody) in Huh7-4P, Huh7-4P-High, and Huh7-4PX cells. To our surprise, the expression levels of these 4 viral proteins were lowest in Huh7-4PX cells compared to parental Huh7-4P and Huh7-4P-High cells (Fig. 4A and B). Of note, the VP35 level in Huh7-4PX cells was particularly low, with only 1% of that in the parental Huh7-4P cells. Next, we reconstituted expression of NP, VP35, and VP30 by lentiviral transduction or L by plasmid transfection in Huh7-4PX cells, respectively. As shown in Fig. 4C to G, the elevated expression of NP, VP30, or L in Huh7-4PX cells had either minor or no inhibitory effect on EBOV-trVLP infection, while the increased VP35 expression displayed the most dramatic inhibitory effect. These results suggested that 4 virial proteins, particularly VP35, had been selected to appropriate levels in Huh7-4PX cells, leading to its exceptional permissiveness for EBOV-SG replication and trVLP propagation.

### Rescue of infectious virions in Huh7-4PX cells using the full-length EBOV genome.

**FIG 5:**
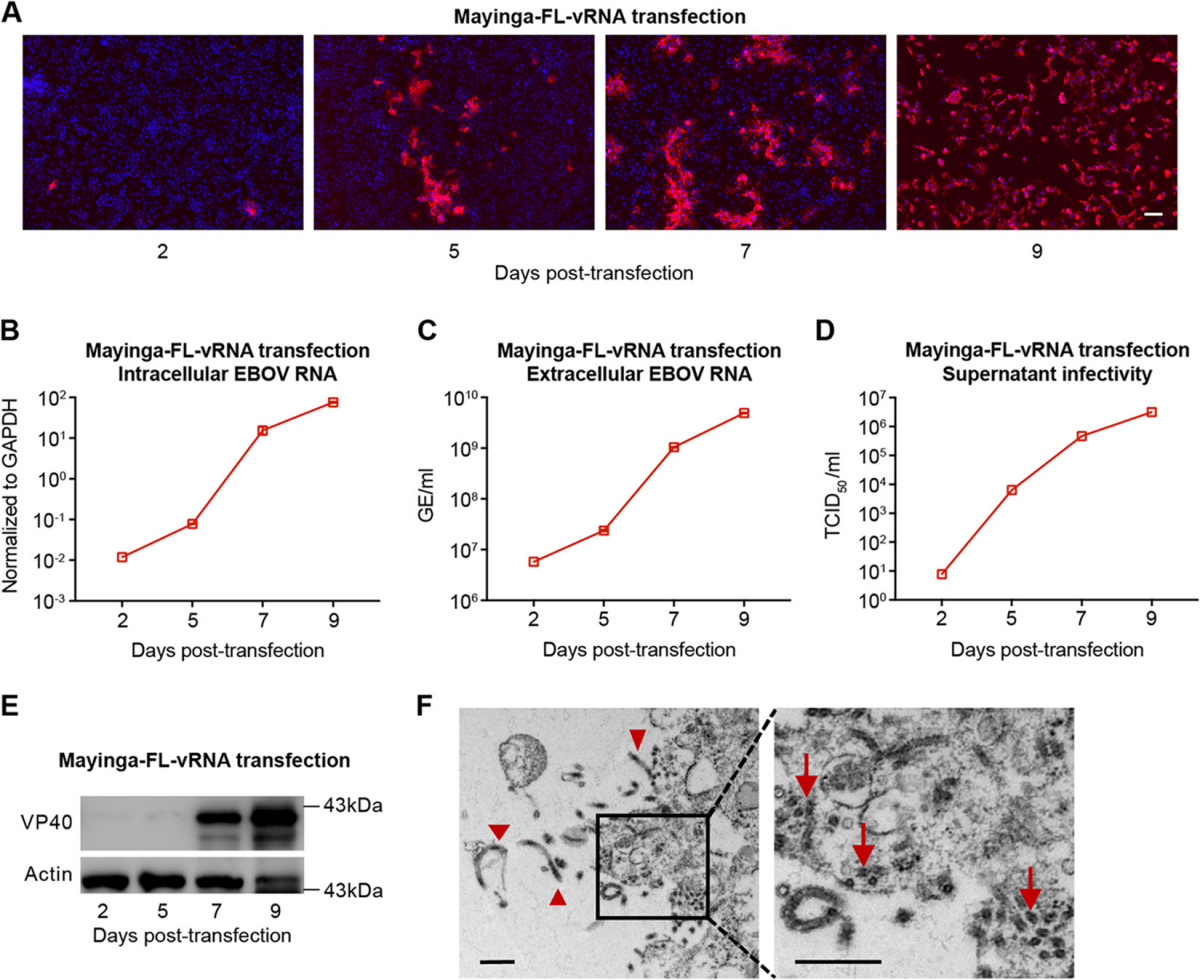
Efficient EBOV rescue in Huh7-4PX cells using the full-length viral genome. Huh7-4PX cells were transfected with vRNA of EBOV (Mayinga isolate) and cultured for 9 days. VP40 immunofluorescence (A), intracellular viral RNA (B), extracellular viral RNA (C), extracellular viral titers (D), and VP40 Western blotting (E) were measured at the indicated time points after transfection. The extracellular EBOV RNA level was indicated as genome equivalents (GE)/ml. Scale bar in panel A, 100 μm. (F) Huh7 cells that had been infected with rescued Mayinga-EBOV at an MOI of 0.1 for 3 days were analyzed by TEM. The filamentous shape of the budding virus (red arrowhead) and the cross-section of the aligned viral nucleocapsid (red arrow) are shown. Scale bar, 2 μm. The data shown in panels B and C are results from 1 of 2 independent experiments and are presented as the mean ± SD of *n* = 2 technical replicates. The data of another experiment are shown in Fig. S4A and B.

We then proceeded to test whether Huh7-4PX could be employed to rescue the fully infectious virions of EBOV. The *in vitro*-transcribed full-length genome (vRNA) of EBOV Yambuku-Mayinga isolate (Mayinga-FL-vRNA) was transfected into Huh7-4PX cells. The number of VP40-positive cells increased rapidly after transfection, and nearly all cells became VP40 positive on day 9 posttransfection when massive cytopathic effect (CPE) was observed (Fig. 5A). Consistently, intracellular (Fig. 5B and Fig. S4A) and extracellular (Fig. 5C and Fig. S4B) viral RNA levels, extracellular viral titers (Fig. 5D), and intracellular VP40 protein level (Fig. 5E) all increased after transfection. TEM of naive Huh7 cells infected with the rescued virus showed the classical filamentous shape of the budding virus and the cross-section of aligned viral nucleocapsid in the cytosol (Fig. 5F). Moreover, Mayinga-EBOV rescued from the RNA-transfection experiment propagated efficiently in naive Huh7 and Vero cells (Fig. S5A and B).

**FIG 6:**
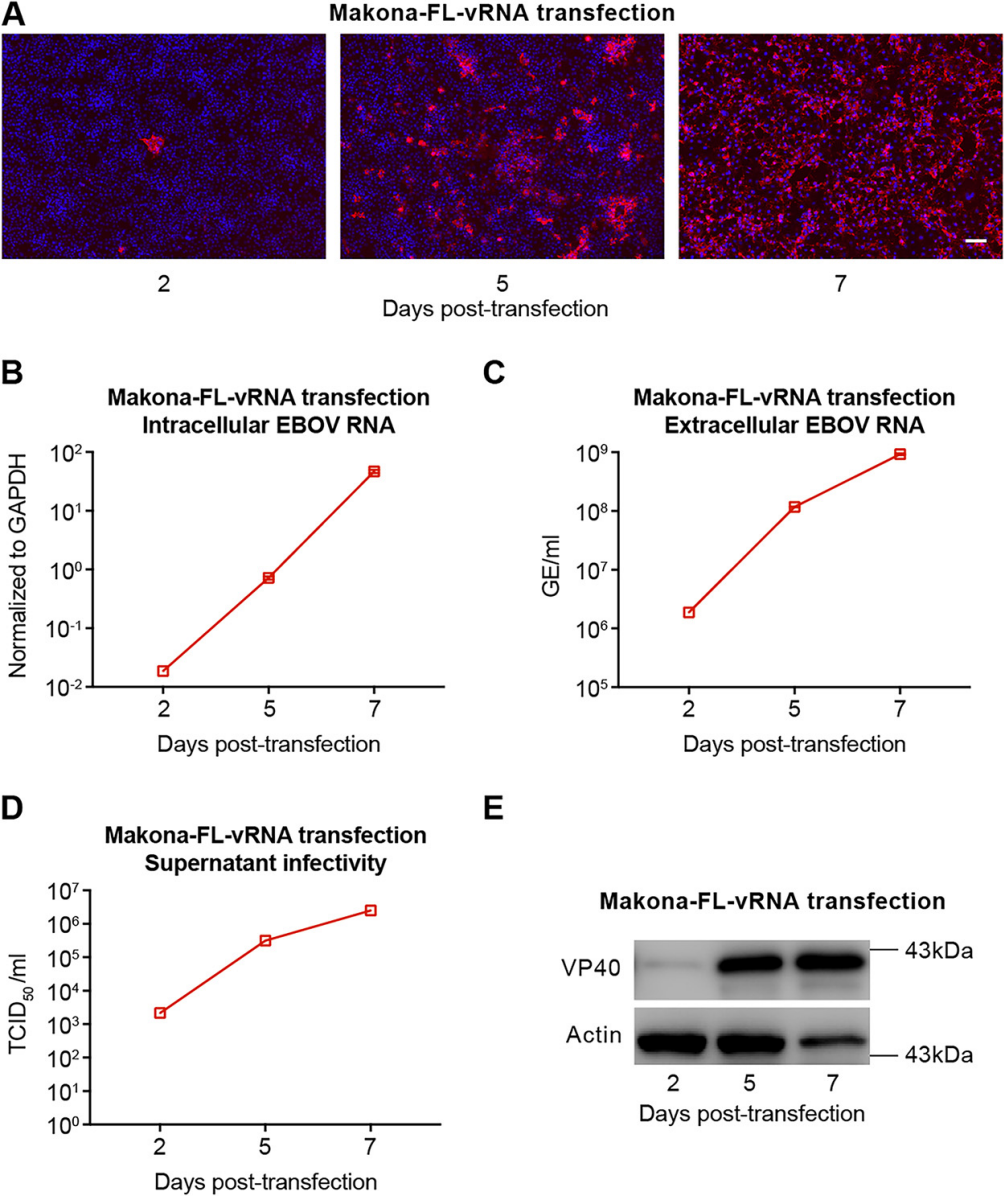
Huh7-4PX cell can be used to rescue EBOV of a heterologous isolate. Huh7-4PX cells were transfected with vRNA of EBOV-Makona isolate and cultured for 7 days. VP40 immunofluorescence (A), intracellular viral RNA levels (B), extracellular viral RNA levels (C), extracellular viral titers (D), and VP40 Western blotting (E) were measured at the indicated time points. The extracellular EBOV RNA level was indicated as genome equivalents (GE)/ml. Scale bar in panel A, 100 μm. The data shown in panels B and C are results from 1 of 2 independent experiments and are presented as the mean ± SD of *n* = 2 technical replicates. The data of another experiment are shown in Fig. S4C and D.

10.1128/mSphere.00235-21.4 FIG S4 Rescue of EBOV-Mayinga or EBOV-Makona isolates by delivering vRNA. Huh7-4PX cells were transfected with vRNA of EBOV-Mayinga (A and B) or EBOV-Makona (C and D). Intracellular viral RNA (A and C) and extracellular viral RNA (B and D) were measured at the indicated time points after transfection. These two experiments are biological replicates of another experiment described in Fig. 5 and Fig. 6, respectively, and the data are presented as the mean ± SD of *n* = 2 technical replicates. Download FIG S4, PDF file, 0.04 MB. Copyright © 2021 Gan et al. 2021 Gan et al. https://creativecommons.org/licenses/by/4.0/ This content is distributed under the terms of the Creative Commons Attribution 4.0 International license.

10.1128/mSphere.00235-21.5 FIG S5 Propagation of rescued EBOV in native cells. (A and B) Huh7 (A) and Vero (B) cells were infected with rescued Mayinga-EBOV at an MOI of 0.1, respectively. Extracellular viral titers were measured at the indicated time points. (C) Huh7 cells were infected with rescued Makona-EBOV at an MOI of 0.1. Extracellular viral titers were measured at the indicated time points. Data shown in panels A to C are results from 2 independent experiments and are presented as the mean ± SD of *n* = 2 biological replicates. Download FIG S5, PDF file, 0.03 MB. Copyright © 2021 Gan et al. 2021 Gan et al. https://creativecommons.org/licenses/by/4.0/ This content is distributed under the terms of the Creative Commons Attribution 4.0 International license.

It has been reported that anti-genomic RNA (cRNA) of a negative-sense RNA virus is preferred over vRNA as the starting material for rescuing virus, possibly to avoid the antisense RNA effects between the introduced vRNA and the mRNA encoding viral proteins transcribed from the transfected plasmids (26). Thus, we also tested this idea in our system by transfection of full-length cRNA of Yambuku-Mayinga isolate (Mayinga-FL-cRNA) into Huh7-4PX cells. As shown in Fig. S6, infectious virions were also successfully recovered.

10.1128/mSphere.00235-21.6 FIG S6 Rescue of EBOV-Mayinga isolate by delivering cRNA. (A to E) Huh7-4PX cells were transfected with anti-genomic RNA (cRNA) of EBOV-Mayinga isolate and cultured for 9 days. VP40 immunofluorescence (A), intracellular viral RNA levels (B), extracellular viral RNA levels (C), extracellular viral titers (D), and VP40 Western blotting (E) were measured at the indicated time points. The intracellular viral RNA level (F) and extracellular viral RNA level (G) of another independent transfection experiment were shown. Scale bar in panel A, 100 μm. Data in panels B, C, F, and G are presented as the mean ± SD of *n* = 2 technical replicates. Download FIG S6, PDF file, 0.5 MB. Copyright © 2021 Gan et al. 2021 Gan et al. https://creativecommons.org/licenses/by/4.0/ This content is distributed under the terms of the Creative Commons Attribution 4.0 International license.

Next, we determined whether Huh7-4PX cells, which stably express NP, VP35, VP30, and L proteins of EBOV-Mayinga isolate, could be utilized to rescue a different EBOV isolate. For this purpose, we chose the EBOV-Makona isolate that has caused the devastating 2013 to 2016 West Africa outbreak. Similar to that of the EBOV-Mayinga isolate, transfection of vRNA of the Makona isolate led to robust production of infectious virions, and nearly all cells became VP40 positive on day 7 posttransfection (Fig. 6A). Intracellular (Fig. 6B and Fig. S4C) and extracellular (Fig. 6C and Fig. S4D) viral RNA levels, extracellular viral titers (Fig. 6D), and intracellular VP40 protein level (Fig. 6E) all rapidly increased after transfection as expected. The rescued Makona-EBOV propagated efficiently in naive Huh7 cells (Fig. S5C). Besides, virus rescue with cRNA of the EBOV-Makona isolate was also successful (Fig. S7). Sequencing analysis of the full-length viral genome showed no mutation in any of the rescued EBOV of Mayinga or Makona from the last day sample of transfection (data not shown), and the 7 uridine-stretch RNA editing site in the GP gene (27) remained unchanged.

10.1128/mSphere.00235-21.7 FIG S7 Rescue of EBOV-Makona isolate by delivering cRNA. (A to E) Huh7-4PX cells were transfected with anti-genomic RNA (cRNA) of EBOV-Makona isolate and cultured for 7 days. VP40 immunofluorescence (A), intracellular viral RNA levels (B), extracellular viral RNA levels (C), extracellular viral titers (D), and VP40 Western blotting (E) were measured at the indicated time points. (F and G) The intracellular viral RNA level (F) and extracellular viral RNA level (G) of another independent transfection experiment are shown. Scale bar in panel A, 100 μm. Data in panels B, C, F, and G are presented as the mean ± SD of *n* = 2 technical replicates. Download FIG S7, PDF file, 0.6 MB. Copyright © 2021 Gan et al. 2021 Gan et al. https://creativecommons.org/licenses/by/4.0/ This content is distributed under the terms of the Creative Commons Attribution 4.0 International license.

## DISCUSSION

In this study, we developed a new reverse genetics system for studying EBOV under different biosafety levels. This system has several following advantages. First, compared to other current EBOV reverse genetics systems that are dependent upon cotransfection of multiple (often 5 to 6) plasmids expressing viral genome and viral proteins, our new system takes advantages of an engineered cell line (Huh7-4PX) in which the 4 viral proteins essential for EBOV replication have been already permanently introduced and their expression levels and stoichiometry fine-tuned to render permissiveness for EBOV replication. Although the full-length EBOV rescue can be also achieved with a decent efficiency using the multiplasmid transfection-based approach that is more flexible in fine-tuning the components of RNP, our system only requires transfection of a single viral RNA genome, which makes experimentations more convenient.

Second, the Huh7-4PX helper cell line can be used to rescue both fully infectious virions and subviral particles. This system is particularly useful to rescue EBOV-trVLP because these subviral particles, once produced, can propagate continuously in the helper cell line. In contrast, propagation of EBOV-trVLP in the previously reported systems requires the repeated cotransfection of the 4 viral proteins essential for EBOV replication. In fact, propagation of EBOV-trVLP in Huh7-4PX cells resembles natural EBOV infection and expansion, rendering a surrogate model to study the entire EBOV life cycle outside BSL-4 conditions. Of note, propagation of EBOV-trVLP in Huh7-4PX cells is considerably less efficient than that of infectious EBOV in Huh7 cells, suggesting that an artificially fixed stoichiometry of RNP components in Huh7-4PX cells may be still less optimal for virion production than the dynamically changing ratio of each RNP component during the natural virus infection.

Third, the Huh7-4PX helper cell can be used to rescue EBOV of a heterologous isolate. Although this finding has been reported before (28), it still has an important implication in the application of the Huh7-4PX helper cell in rescuing novel EBOV isolates because it alleviates the needs to construct another Huh7-4PX helper cell that is customized for a novel EBOV isolate, which could be a time-consuming process. Interestingly, we found that the Huh7-4PX cell was able to, albeit at a lower efficiency, support the replication and transcription of the minigenome of two Marburg virus (MARV) isolates (Angola-2005 isolate and Musoke isolate) (Fig. S8). This result raises an exciting possibility that the Huh7-4PX cell may have a broader application in developing reverse genetics systems for other filoviruses of different species or even different genera. More future investigations are warranted to determine the potential expanded application of the Huh7-4PX cell.

10.1128/mSphere.00235-21.8 FIG S8 Huh7-4PX cells support replication of Marburg virus minigenome. (A) Schematics of EBOV-MG and MARV-MG with a EGFP-NanoLuc (NLuc) reporter. (B) Huh7 and Huh7-4PX cells that had been transfected with EBOV-MG RNA or MARV-MG (Angola-2005 isolate, GenBank accession no. DQ447653; Musoke isolate, GenBank accession no. NC001608) RNA, respectively, for 2 days were analyzed for luciferase activity. Data are presented as the mean ± SD of *n* = 3 biological replicates. Download FIG S8, PDF file, 0.04 MB. Copyright © 2021 Gan et al. 2021 Gan et al. https://creativecommons.org/licenses/by/4.0/ This content is distributed under the terms of the Creative Commons Attribution 4.0 International license.

There are also some limitations to our system. First, virus rescue and trVLP production are restricted to Huh7 cells. It remains a question whether such a helper cell can be developed using other cell lines. Second, the four viral nucleocapsid protein-coding genes have been stably integrated into the Huh7-4PX cells, making it impossible to use this model to study any mutations within these four viral proteins. In addition, because the expression levels and stoichiometry of the four viral RNP proteins are fixed in the cells, it remains to be determined how it would affect the EBOV life cycle, during which their expressions are believed to be dynamic.

We have explored potential mechanisms why the Huh7-4PX cell is competent in supporting EBOV replication. To our surprise, compared to its parental cells, the Huh7-4PX cell has the lowest expression level of NP, VP35, VP30, and L proteins. Among the four viral proteins, VP35 is most critical. Its expression level in the Huh7-4PX cell has been decreased to a greater degree than others, and its overexpression in the Huh7-4PX cell has the greatest inhibitory effect on EBOV replication. It has been reported that excess VP35 inhibits viral replication by affecting the formation of replication complex (18), which may at least partially explain why VP35 expression level is so critical in Huh7-4PX cells. It would be interesting to further understand the mechanism and to develop new antivirals that could mimic the inhibitory function of VP35. It would also be worth investigating whether the VP35-mediated inhibition could be rescued by overexpression of other viral proteins, which may help understand the regulation of the virus replication complex. As an immune antagonist, VP35 blocks virus-mediated induction of interferon signaling response (29), while VP24 inhibits downstream signaling upon the interferon treatment (30). The potential contribution of VP35-antagonizing interferon signaling to the permissiveness of Huh7-4PX cells needs to be further investigated.

The rescue of negative-sense RNA viruses has lagged much behind positive-sense RNA viruses largely due to the prerequisite to deliver viral ribonucleoproteins alongside the viral genome. We adopted the advantageous method of positive-sense RNA virus rescue and simplified EBOV rescue by directly delivering viral genome into cells. Consistent with a previous study (31), we found that both vRNA and cRNA can be used to rescue EBOV, challenging conventional wisdom in the field that cRNA may be preferred over vRNA in rescuing negative-sense RNA viruses (32–35). In addition, delivery of vRNA, the viral genome, should recapitulate the early events of EBOV life cycle in a more genuine manner. Further investigations are needed to determine which form of RNA is more efficient in rescuing EBOV.

## MATERIALS AND METHODS

### Cell culture.

Human embryonic kidney cells (HEK293T), African green monkey kidney cells (Vero), human hepatoma cells (Huh7) (obtained from National Collection of Authenticated Cell Cultures, Chinese Academy of Sciences), and their derivative cell lines were maintained in Dulbecco’s modified Eagle medium (DMEM) (Thermo Fisher Scientific) supplemented with 10% fetal bovine serum (FBS), 10 mM HEPES buffer, 100 U/ml penicillin-streptomycin, 10 mM l-glutamine, and 0.1 mM nonessential amino acids (Thermo Fisher Scientific) at 37°C in a moist environment containing 5% CO_2_. To subclone Huh7-4P-High cells, the cells were trypsinized and resuspended in complete culture medium at 1 × 10^4^ cells/ml as the starting concentration. We added 200 μl of the cell suspension to the first well of a 96-well plate, and then 2-fold serial dilutions were conducted across the entire plate. The final medium volume of each well was supplemented to 200 μl. After culturing for weeks, monoclonal cells were trypsinized and seeded back into a 96-well plate for proliferation. The viability, growth kinetics, and morphology of Huh7-4P, Huh7-4P-High, and Huh7-4PX cells were like those of Huh7 cells.

### Construction of plasmids.

The full-length genome of EBOV Yambuku-Mayinga isolate (NC002549) and Makona-C07 isolate (GenBank accession no. KJ660347) were synthesized in several fragments by GenScript. To construct EBOV MG, SG, and full-length genome (FLG) plasmids, PCR was carried out with Q5 High-Fidelity DNA polymerase (New England BioLabs), and products were assembled with the NEBuilder HiFi DNA Assembly cloning kit (New England BioLabs). DNA was transformed into DH5α or TOP10 competent cells (Tiangen). Plasmid DNA was purified with the TIANprep mini plasmid kit (Tiangen) or the NucleoBond Xtra Midi Plus kit (Macherey-Nagel). All the genome sequences were fused between a T7 promoter and a hepatitis D virus ribozyme followed by a T7 terminator as previously reported (36). All constructs were verified by Sanger sequencing.

### Viruses.

Viruses used in this study were all recombinants produced by reverse genetics. Rescue of viruses was achieved by electroporation of the corresponding *in vitro*-transcribed full-length viral genome RNA into Huh7-4PX cells. All experiments involving live EBOV were conducted in the BSL-4 facility of Wuhan National Biosafety Laboratory, Chinese Academy of Sciences.

### *In vitro* transcription.

*In vitro* transcription of EBOV MG, SG, and FLG was performed using the MEGAscript T7 transcription kit (Thermo Fisher Scientific) following the manufacturer’s protocol. Following 4.5 h incubation at 37°C, the sample was treated with 1 μl of TURBO DNase for 15 min at 37°C, precipitated with 30 μl of lithium chloride, and chilled for at least 1 h at −20°C. RNA was then pelleted at 13,200 × *g* for 10 min and washed once with 1 ml of 75% ethanol. After thorough removal of ethanol, RNA was air-dried at room temperature for 2 min and resolved in 100 μl of RNase-free water.

### RNA electroporation.

Cells were trypsinized, resuspended in complete growth medium, and pelleted at 260 × *g* for 5 min. Cells were then washed once with 5 ml of Opti-MEM (Thermo Fisher Scientific) and resuspended in Opti-MEM at a concentration of 1.5 × 10^7^ cells/ml. We mixed 400 μl of the cell suspension thoroughly with 10 μg of *in vitro*-transcribed RNA in a 0.4-cm electroporation cuvette (Bio-Rad). Electrical pulse was exerted once at 270 V, 950 μF, and 100 Ω with the Gene Pulser Xcell electroporation system (Bio-Rad). The electroporated cells were immediately transferred into 10 ml of complete growth medium in a 15-ml Falcon tube. The cell suspension was transferred to a 10-cm dish or T75 flask for normal culturing and was usually passaged at a 1:5 ratio every 3 days. All the supernatant, RNA, protein, and cell samples were harvested during each cell passage with consistent amounts.

### Antibodies.

Rabbit polyclonal antibodies against EBOV NP, VP35, and VP30 proteins have been described previously (16). Mouse polyclonal antibody against EBOV VP40 protein was a gift from Zhong Huang (Institut Pasteur of Shanghai). Mouse monoclonal antibody against β-actin (catalog no. AM1021B) was bought from Abgent. The following secondary antibodies were used: donkey anti-mouse Alexa Fluor 555 conjugate (catalog no. A31570; Thermo Fisher Scientific), goat anti-mouse Alexa Fluor 555 conjugate (catalog no. A21422; Thermo Fisher Scientific), goat anti-mouse horseradish peroxidase (HRP) conjugate (catalog no. 115-035-003; Jackson ImmunoResearch), and goat anti-rabbit HRP conjugate (catalog no. 31460; Thermo Fisher Scientific).

### Western blotting.

Cell lysates were prepared by lysis in 1× SDS loading buffer (62.5 mM Tris-HCl [pH 6.8], 10% [wt/vol] SDS, 50% glycerol, 50 mM dithiothreitol [DTT], 0.05% [wt/vol] bromophenol blue, and 3.33% β-mercaptoethanol) after removal of cell culture medium. The lysates were incubated at 98°C for 10 min, centrifuged, homogenized, and then loaded on 10% PAGE gels. Transferred polyvinylidene difluoride (PVDF) membrane was probed with primary antibodies for 2 h, followed by incubation with the HRP-conjugated secondary antibodies for 1 h. Chemiluminescence was then developed by incubation of the membrane with the Immobilon Western chemiluminescent HRP substrate (Millipore) and scanned with the Amersham Imager 600 (General Electric). Protein quantification was processed with ImageJ software and normalized against internal actin.

### Immunofluorescence assay.

Cells seeded in 96-well plates were fixed with 4% of paraformaldehyde for at least 30 min and permeabilized and blocked for 1 h by blocking buffer (0.3% Triton X-100, 3% bovine serum albumin [BSA] [wt/vol], and 10% FBS in phosphate-buffered saline [PBS]) before 1 h of incubation with primary antibody prepared in binding buffer (0.3% Triton X-100, 3% BSA in PBS). Cells were then stained for 1 h with secondary antibody prepared in binding buffer before imaging. Images were acquired and processed with an IX73 inverted microscope (Olympus).

### Luciferase assay.

*Gaussia* luciferase activity was measured with the BioLux *Gaussia* luciferase assay kit (New England BioLabs). A 50-μl volume of cleared culture supernatant was added into a 96-well white plate, and 20 μl of GLuc assay solution was prepared and thoroughly mixed with the sample immediately before luminescence detection with a Synergy H1 reader (Biotek). NanoLuc activity was measured with Nano-Glo luciferase assay system (Promega). A 25-μl volume of cleared culture supernatant was added into a 96-well white plate, and 25 μl of NanoLuc assay solution was prepared and thoroughly mixed with the sample before luminescence detection with a Synergy H1 reader.

### RNA isolation, reverse transcription, and quantification.

Cellular and supernatant RNA was isolated with the TRIzol reagent (Thermo Fisher Scientific) or RNA Lyzol reagent (ExCell Bio). For supernatant, 100 μl was harvested with 1 ml of TRIzol reagent. Reverse transcription was performed using the ReverTra Ace quantitative PCR (qPCR) RT kit (Toyobo) following the protocol of the manufacturer. Quantitative PCR was carried out utilizing the SYBR green real-time PCR master mix (Toyobo) and analyzed with QuantStudio 6 Flex real-time PCR system (Thermo Fisher Scientific). Sequences of quantitative PCR primers were as follows: *EBOV* forward primer, 5′-GGGAATGGAGTGGCAACTGA-3′; *EBOV* reverse primer, 5′-GCTGCTGGTAGACACTCACT-3′; *EBOV L* forward primer, 5′-CGCCAGTCCCGAACACAGAC-3′; *EBOV L* reverse primer, 5′-GCAATCCCGAATGTGGTAGAACC-3′; human glyceraldehyde-3-phosphate dehydrogenase (*GAPDH*) forward primer, 5′-GAAGGTGAAGGTCGGAGTC-3′; human *GAPDH* reverse primer, 5′-GAAGATGGTGATGGGATTTC-3′; *EGFP* forward primer, 5′-CCACATGAAGCAGCACGACT-3′; and *EGFP* reverse primer, 5′-GCTCGATGCGGTTCACCAG-3′.

### Antiviral assays.

Neutralizing antibody c2G4 (provided by Yi Shi, Institute of Microbiology, Chinese Academy of Sciences) was serially diluted to the indicated concentrations and incubated with EBOV-trVLP for 1 h at 37°C and used to infect Huh7-4PX cells. The cells were fixed at 48 h postinfection, and VP40-positive foci were analyzed by immunofluorescence. Remdesivir (MedChemExpress) was serially diluted to the indicated concentrations and mixed with EBOV-trVLP to infect Huh7-4PX cells. The cell lysates were harvested at 72 h posttreatment for measuring RNA levels. IFN-α (Roche), IFN-γ (R&D Systems), and IFN-λ3 (R&D system) were serially diluted to the indicated concentrations and added to Huh7-4PX cells 12 h before EBOV-trVLP infection. The cell lysates were harvested at 72 h posttreatment for measuring RNA levels.

### Lentivirus package.

We cotransfected 7 × 10^5^ HEK293T cells in a 6-well plate with 2 μg of lentiviral vector pLVX expressing target gene, together with 1.5 μg of packaging plasmid pCMV-dR8.91 and 1 μg of pCMV-VSV-GP plasmid using polyethylenimine (PEI) (Polysciences). The culture supernatants were refreshed 6 h posttransfection and harvested 48 h posttransfection and filtered through 0.45-μm-pore-size filters (Millipore).

### Overexpression of EBOV proteins in Huh7-4PX cells.

Huh7-4PX cells were infected with NP, VP35, or VP30 lentivirus, respectively, for 24 h. Transduced cells were passaged and cultured for another 72 h before infection with EBOV-trVLP. The cell lysates were harvested at 72 h postinfection for measuring protein and RNA levels. For L overexpression, Huh7-4PX cells were transfected with pcDNA3.1 plasmid encoding L protein. Transfected cells were seeded 24 h posttransfection and then infected with EBOV-trVLP. The cell lysates were harvested at 72 h postinfection for measuring protein and RNA levels.

### Flow cytometry.

Cells were trypsinized, resuspended in complete growth medium, and pelleted at 260 × *g* for 5 min. Cells were washed once with PBS and resuspended in sorting buffer (PBS containing 1% newborn bovine serum, 2.5 mM EDTA, and 10 μg/ml gentamicin). Cell sorting was done with MoFlo Astrios (Beckman Coulter). After debris and nonsinglet events exclusion, a 488-nm laser was used to excite GFP signal, and 0.57% of cells with the highest signal were sorted out. After sorting, cells were pelleted at 260 × *g* for 5 min and resuspended with complete medium before seeding into a 96-well plate. Cytometry data were analyzed and processed with FlowJo software (FlowJo LLC).

### Titration of EBOV-trVLP.

Huh7-4PX cells were seeded into 96-well plates at 1 × 10^4^ cells/well. trVLP samples were serially diluted 10-fold in complete medium and used to infect the cells (3 replicates per dilution). Following 2 days of incubation, the cells were fixed and immunostained as described above. The trVLP titer is expressed as focus-forming units per milliliter of supernatant (FFU/ml).

### Purification of EBOV-trVLP.

Culture medium containing trVLP was centrifuged for 20 min at 3,000 × *g* to remove cell debris and then ultracentrifuged at 100,000 × *g* for 2 h. The pellet was completely resuspended, homogenized, and transferred for purification by fractionation with iodixanol. The resuspension was centrifuged at 150,000 × *g* for 16 h. Fractions were taken out for RNA quantity and infectivity titer measurements, and the best fraction was used for negative staining with 2% uranyl acetate and processed for transmission electron microscopy (TEM) under an FEI Tecnai G2 20 Twin electron microscope (Thermo Fisher Scientific).

### Titration of EBOV.

Huh7 cells were seeded into 96-well plates at 4 × 10^4^ cells/well. Virus samples were serially diluted 10-fold in 3% FBS medium and used to infect the cells (8 wells per dilution). Following 6 days of incubation, the cells were fixed and immunostained as described above. The viral titer is expressed as 50% tissue culture infectious dose per milliliter of supernatant (TCID_50_/ml).

### Reverse transcriptase PCR and sequencing.

Supernatant RNA samples were reverse transcribed with the HiScript 1st Strand cDNA synthesis kit (Vazyme) following the manufacturer’s protocol. Nested PCR was then performed with PrimeStar GXL DNA polymerase (TaKaRa) to amplify the cDNA in 10 segments, and DNA products were sent for sequencing. Primer sequences are available upon reasonable request.

### TEM of infected cells.

Ultrathin-section electron microscopy was performed as described previously (6). Briefly, Huh7 cells were cultured in 0.4-μm-pore-size Transwells (Corning), in which 2 × 10^5^ cells were seeded on polycarbonate membrane for each chamber and infected with EBOV at an MOI of 0.1. At 72 h after infection, cells were fixed with 4% of paraformaldehyde for at least 30 min and stored with the fix buffer at 4°C overnight. Cells and polycarbonate membranes were removed from Transwell chambers as a complete monolayer. The cell monolayer was postfixed with 1% osmium tetroxide in 0.1 M PBS for 30 min and dehydrated with a series of ethanol gradients followed by propylene oxide before being embedded in resin. Thin sections were stained with 2% uranyl acetate and Reynolds lead citrate for 15 min and processed for TEM imaging under an FEI Tecnai G2 20 Twin electron microscope.

### Statistical analysis.

Prism 7 (GraphPad Software) was used for statistical analysis. One-way analysis of variance (ANOVA) (Kruskal-Wallis test) was used to evaluate the efficacy of IFNs on EBOV-trVLP and to compare the effects of excess viral protein on EBOV-trVLP infection. A *P* value of <0.05 was considered to indicate a statistically significant difference.
