# Supplementary figures and images for: Development of a New Reverse Genetics System for Ebola Virus
Source: mSphere. 2021 May 5;6(3):e00235-21. doi: 10.1128/mSphere.00235-21 (PMC8103987; doi:10.1128/mSphere.00235-21)

**Figure S1**

**A**

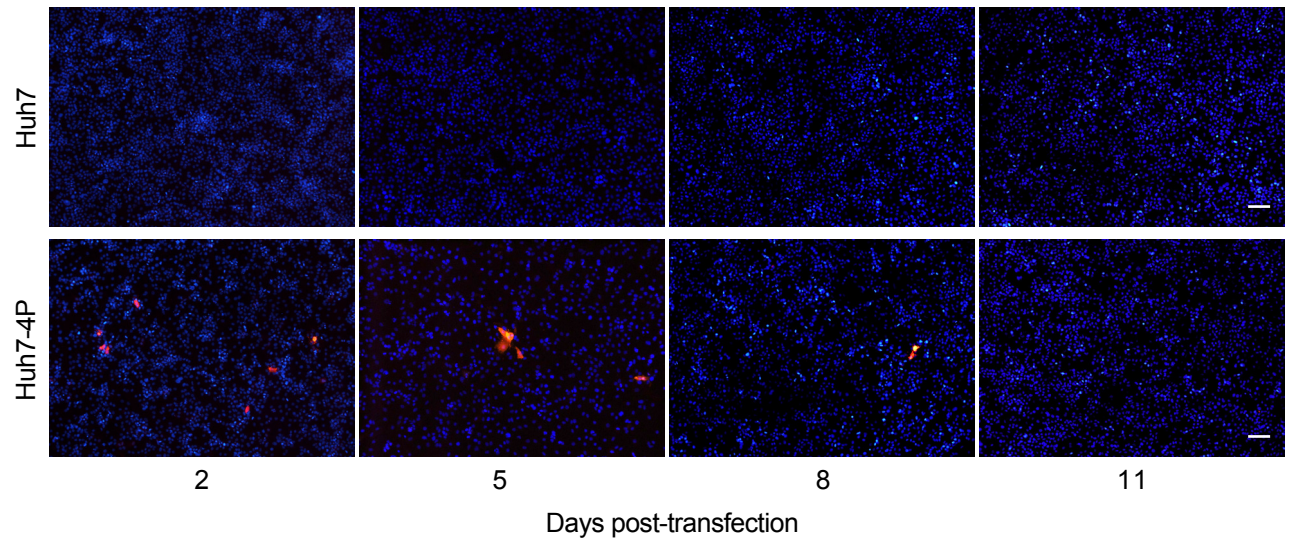

**B**

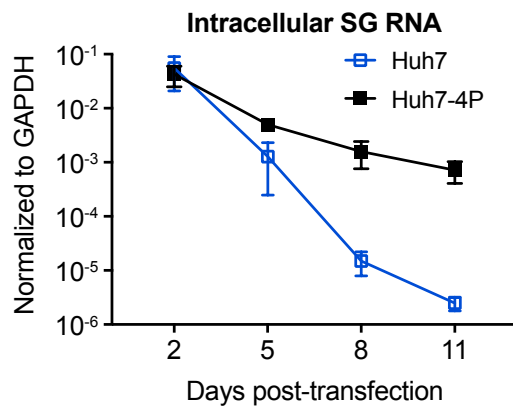

**C**

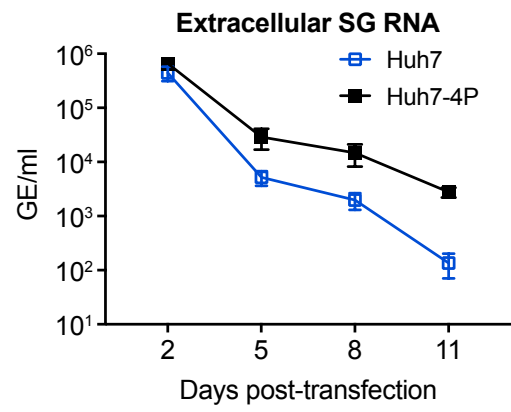

Supplement: FIG S1 [file mSphere.00235-21-sf001.pdf]

Figure S2

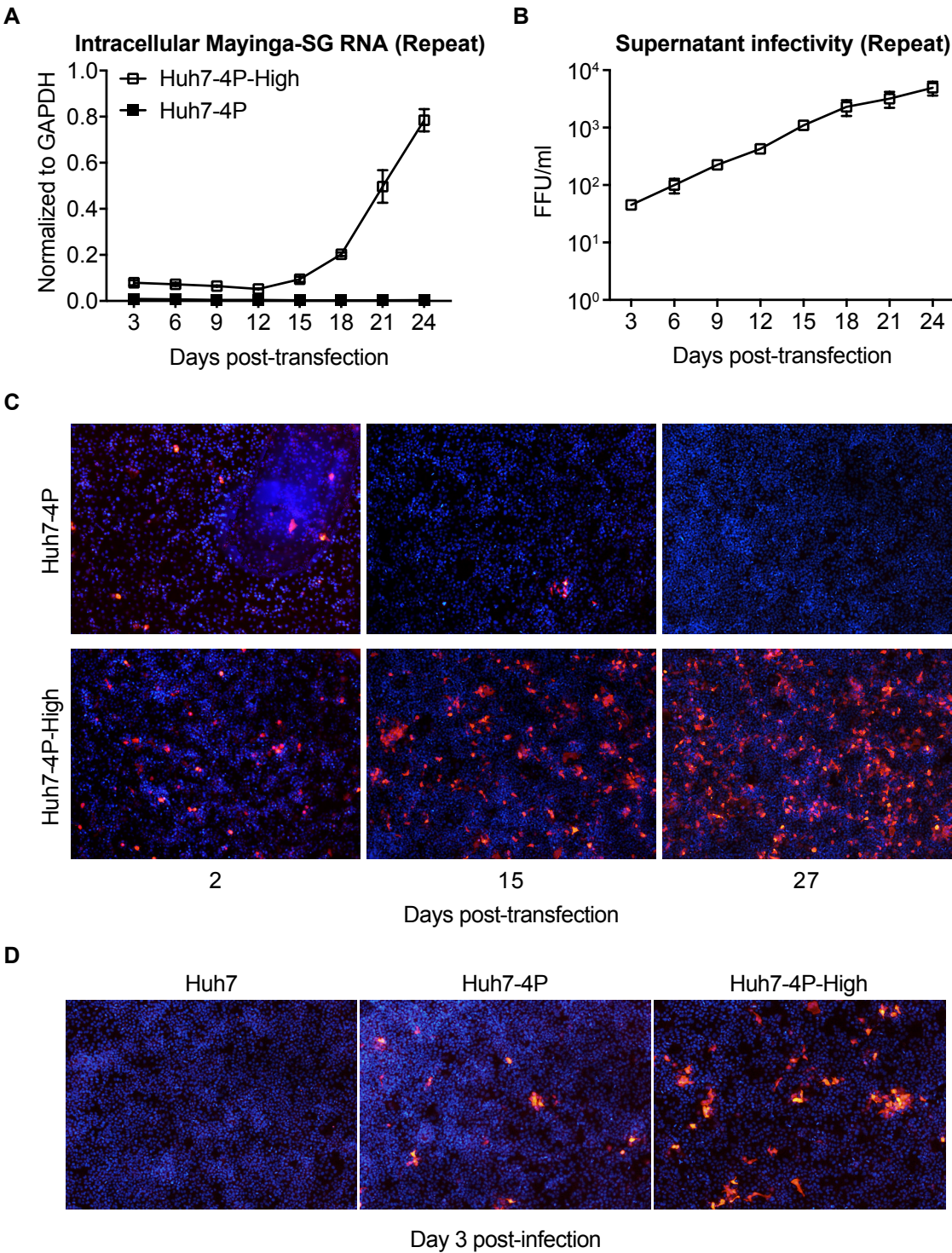

Supplement: FIG S2 [file mSphere.00235-21-sf002.pdf]

Figure S3

A

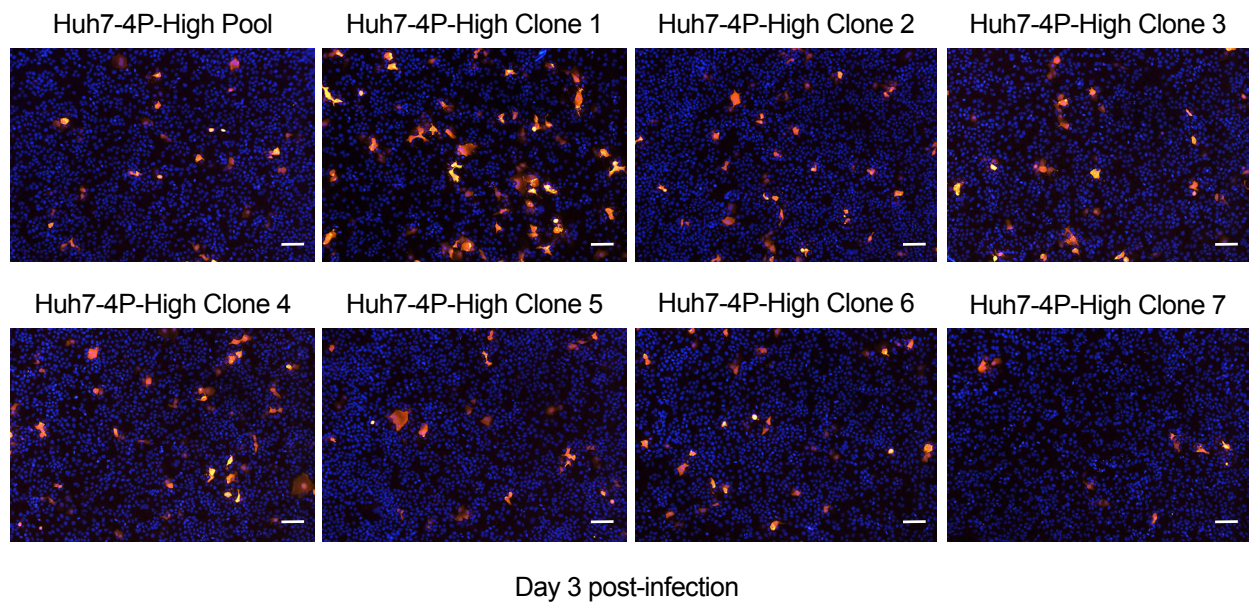

B

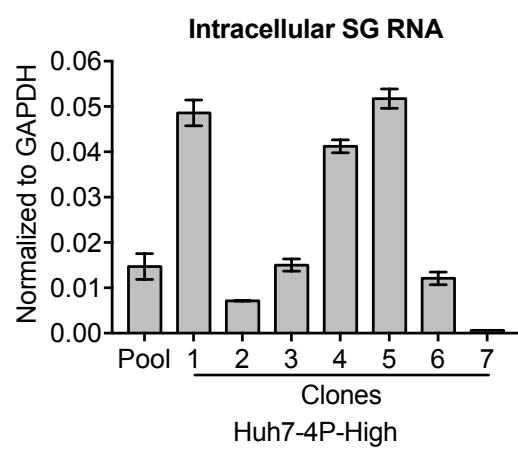

C

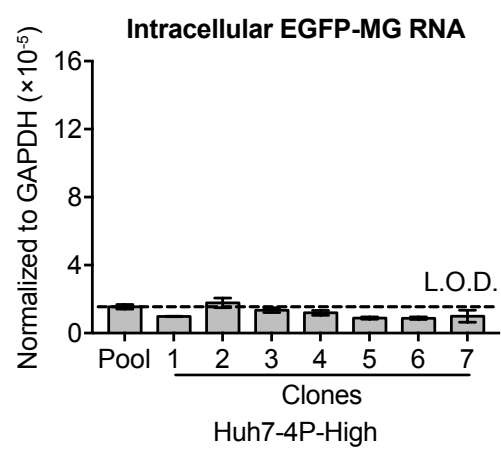

Supplement: FIG S3 [file mSphere.00235-21-sf003.pdf]

**Figure S4**

**A**

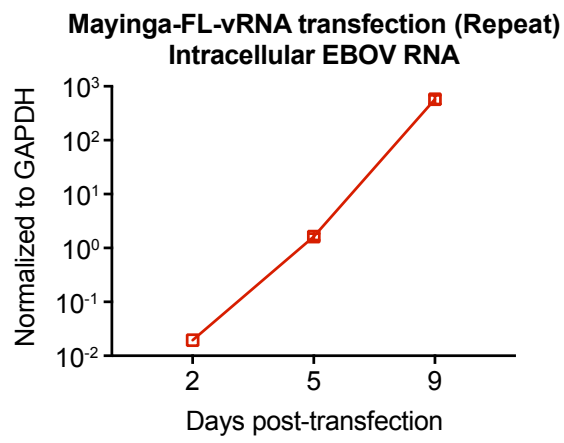

**B**

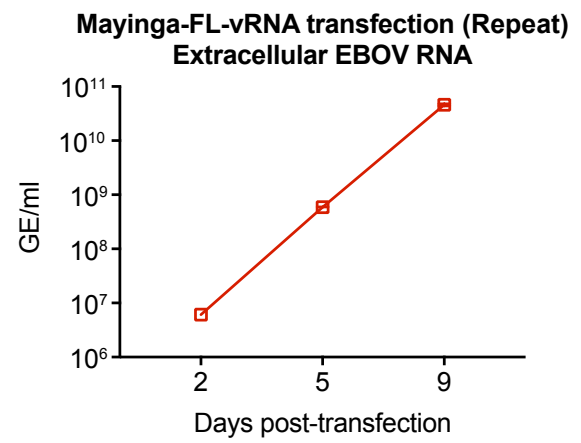

**C**

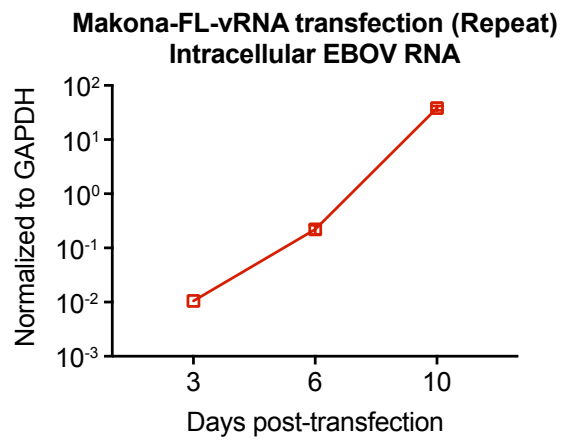

**D**

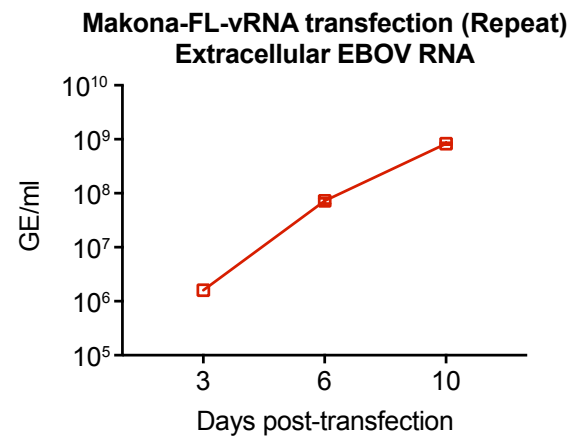

Supplement: FIG S4 [file mSphere.00235-21-sf004.pdf]

**Figure S5**

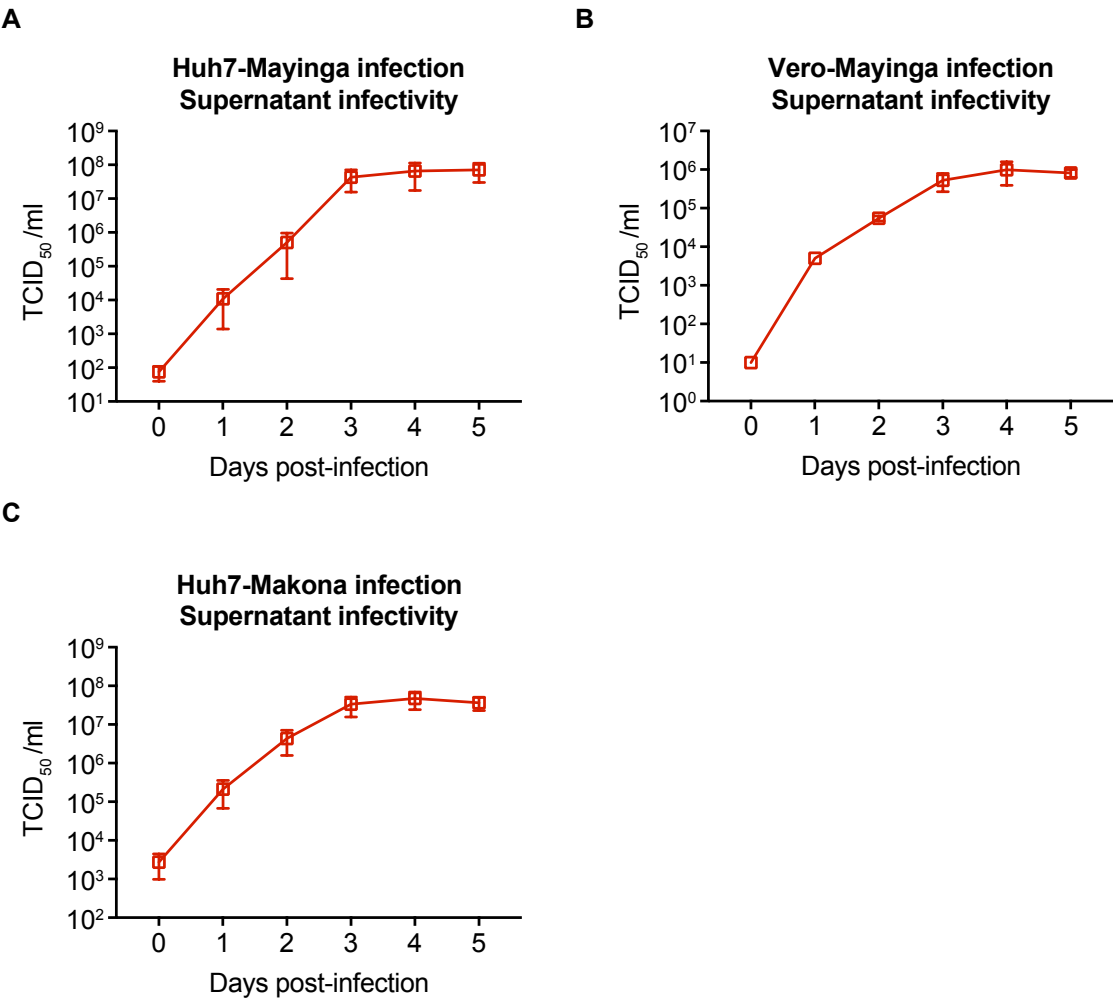

Supplement: FIG S5 [file mSphere.00235-21-sf005.pdf]

**Figure S6**

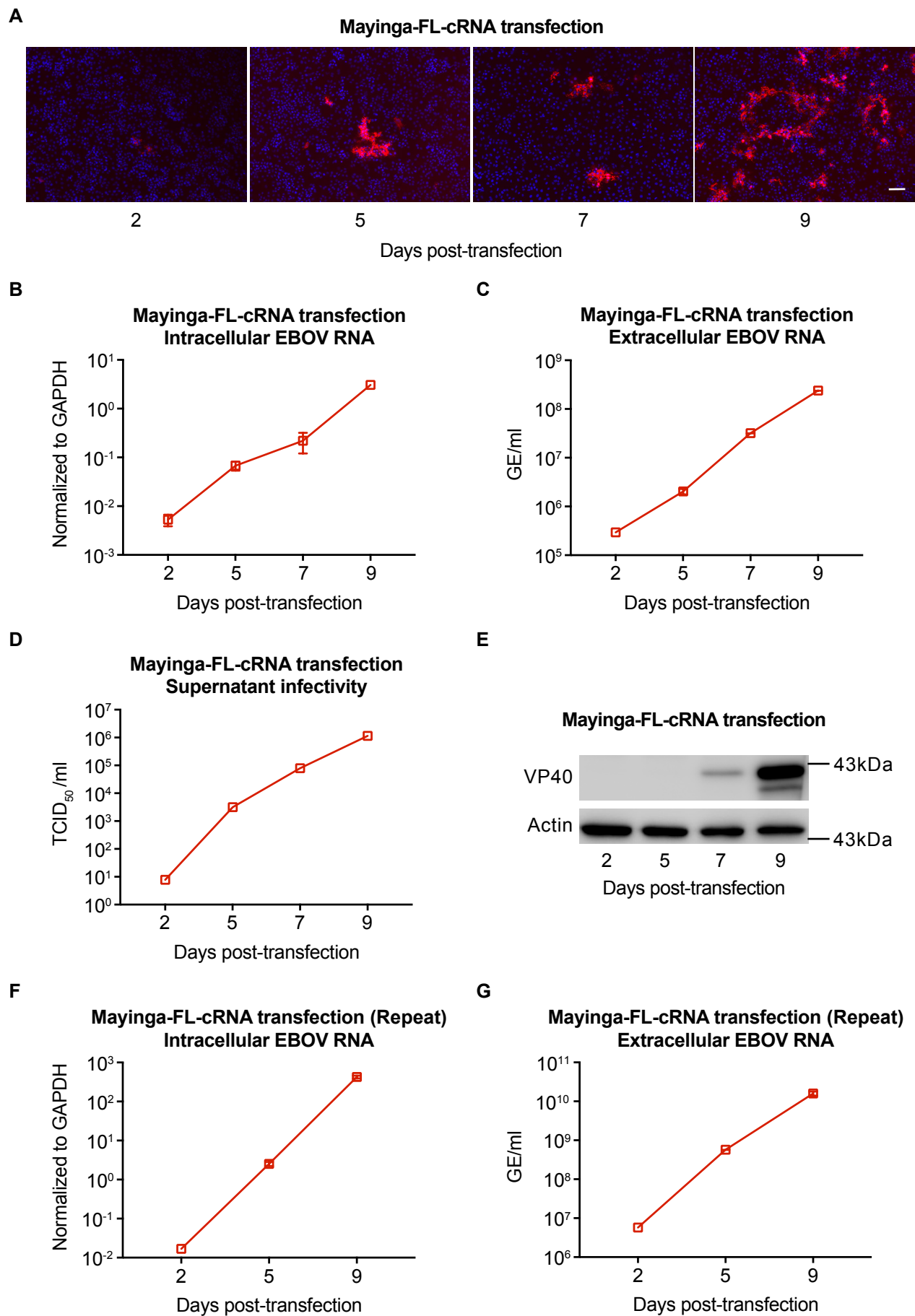

Supplement: FIG S6 [file mSphere.00235-21-sf006.pdf]

**Figure S7**

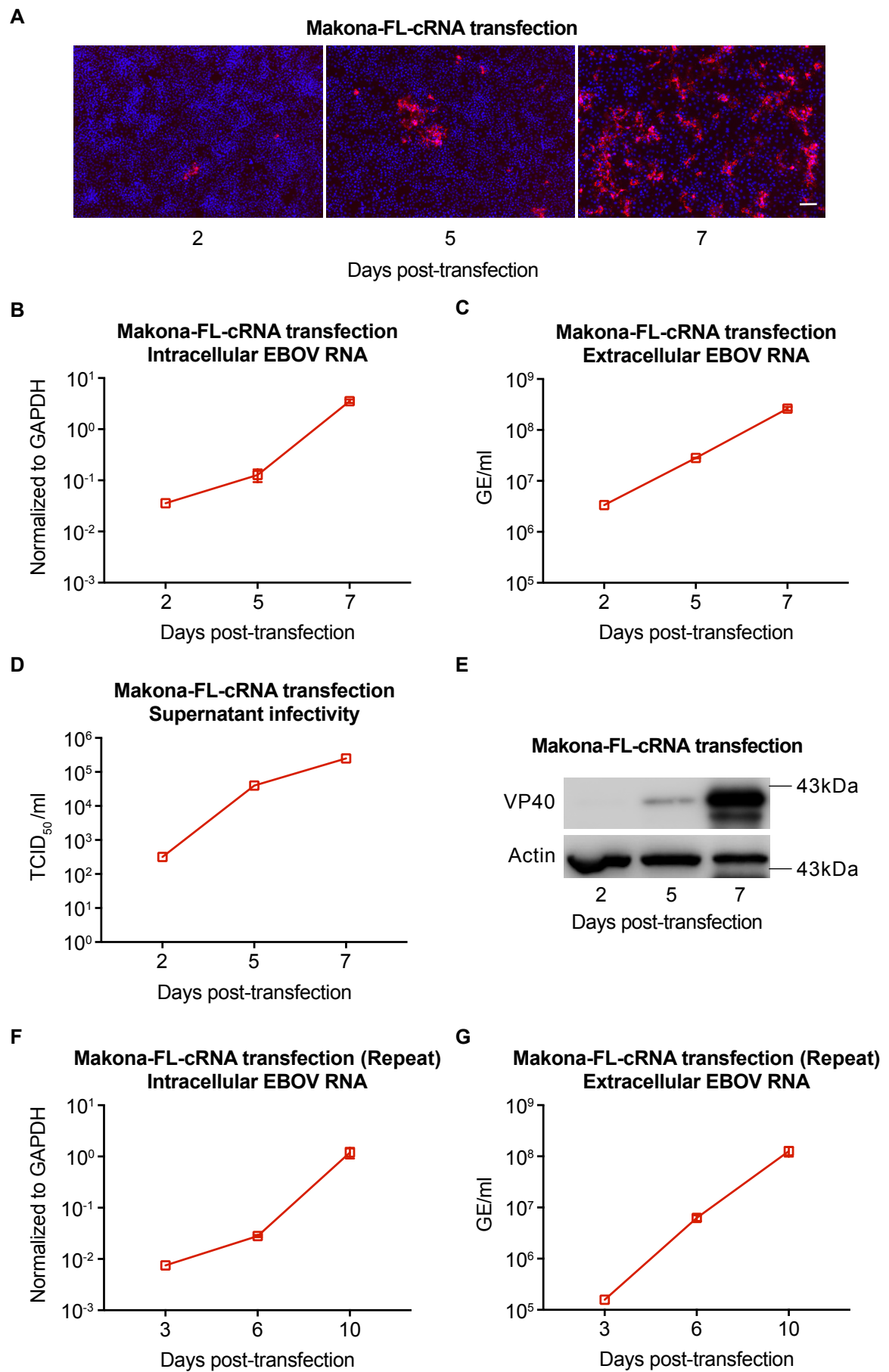

Supplement: FIG S7 [file mSphere.00235-21-sf007.pdf]

**Figure S8**

**A**

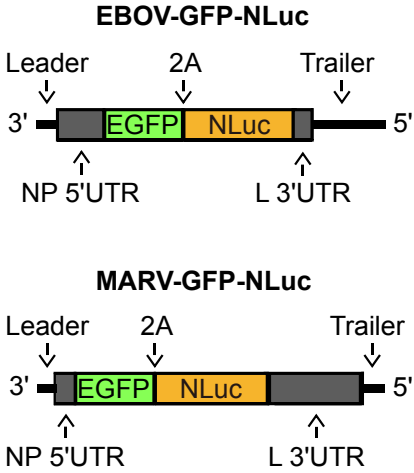

**B**

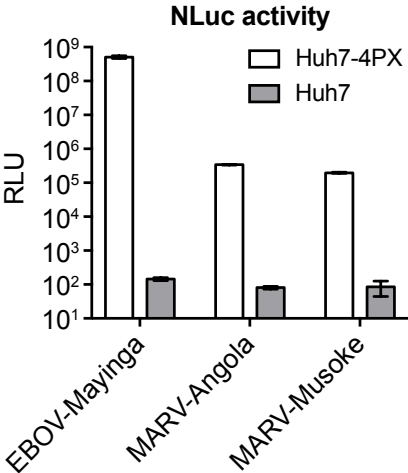

Supplement: FIG S8 [file mSphere.00235-21-sf008.pdf]
